# East Tennessee Dental Association

**Published:** 1868-02

**Authors:** S. H. Smith


### EAST TENNESSEE DENTAL ASSOCIATION.

On the third Thursday of October last, a number of Dentists from different parts of East Tennessee, met at the office of Dr. J. Fouche, in Knoxville, for the purpose of organizing an Association. At a preliminary meeting held some weeks before, a committee had been appointed to draft a Constitution to be presented at this meeting.

Dr. Carson, of Rhea county, the chairman, called the meeting to order.

Dr. W. H. Cooke, of Cleveland, reported a Constitution, which after considerable discussion was adopted.

An election of officers for the ensuing year resulted in the choosing of Dr. J. Fouche, of Knoxville, President; Dr. W. H. Cooke, of Cleveland, Recording Secretary and Treasurer; and Dr. S. H. Smith, of Knoxville, Corresponding Secretary.

The annual meetings will be held at Knoxville on the third Wednesday of October.

Two other meetings in the year are provided for. This year they will be held on the third Wednesday in March at Cleveland, and on the third Wednesday of June at Morristown.

Such an organization has been long needed in East Tennessee. It is only necessary that the profession in East Tennessee sustain it, to make it of very great advantage to themselves and the community.

S. H. SMITH, *Cor. Secretary.*

